# The Role of Seaweed Polysaccharides in Gastrointestinal Health: Protective Effect against Inflammatory Bowel Disease

**DOI:** 10.3390/life13041026

**Published:** 2023-04-16

**Authors:** N. M. Liyanage, D. P. Nagahawatta, Thilina U. Jayawardena, You-Jin Jeon

**Affiliations:** 1 Department of Marine Life Sciences, Jeju National University, Jeju 690-756, Republic of Korea; 2 Department of Chemistry, Biochemistry and Physics, Université du Québec à Trois-Rivières, Trois-Rivières, QC G8Z 4M3, Canada; 3 Marine Science Institute, Jeju National University, Jeju 63333, Republic of Korea

**Keywords:** seaweed, polysaccharide, inflammatory bowel disease, gastrointestinal health

## Abstract

Inflammatory bowel disease (IBD) is a prominent global public health issue. Anti-inflammatory medications, immunosuppressants, and biological therapies are currently used as treatments. However, they are often unsuccessful and have negative consequences on human health. Thus, there is a tremendous demand for using natural substances, such as seaweed polysaccharides, to treat IBD’s main pathologic treatment targets. The cell walls of marine algae are rich in sulfated polysaccharides, including carrageenan in red algae, ulvan in green algae, and fucoidan in brown algae. These are effective candidates for drug development and functional nutrition products. Algal polysaccharides treat IBD through therapeutic targets, including inflammatory cytokines, adhesion molecules, intestinal epithelial cells, and intestinal microflora. This study aimed to systematically review the potential therapeutic effects of algal polysaccharides on IBD while providing the theoretical basis for a nutritional preventive mechanism for IBD and the restoration of intestinal health. The results suggest that algal polysaccharides have significant potential in complementary IBD therapy and further research is needed for fully understanding their mechanisms of action and potential clinical applications.

## 1. Introduction

The intestine is the largest surface that creates a connection between the body and the external environment. In addition to being a significant entry point for dangerous infections, it carries food antigens as well as a large, varied bacterial flora that must be tolerated. The collaboration of several regulatory systems stops the immune system from responding toward innocuous external antigens found in the colon, and it maintains the balance between the host and flora [1]. Intestinal inflammation occurs due to the increase in proinflammatory stimuli, the disruption of the intestinal barrier, defective immunoregulatory mechanisms, or excessive immune effector activity [2]. In humans, the most frequent cause of chronic intestinal inflammation is inflammatory bowel disease (IBD) [3].

**Figure 1:**
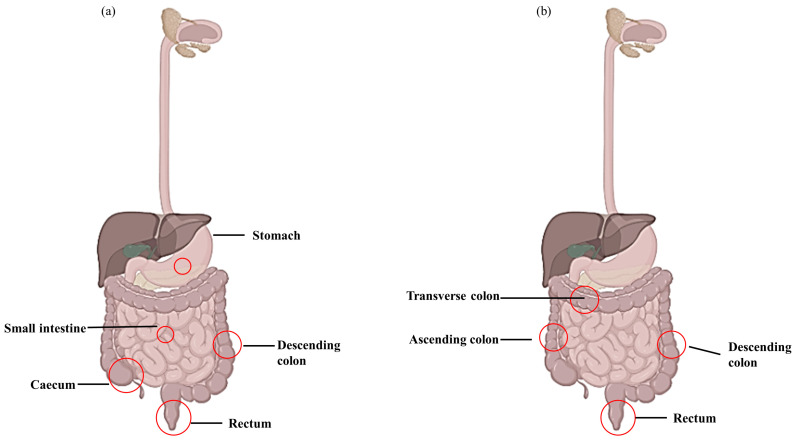
Lesions appear during Crohn’s disease (CD) and ulcerative colitis (UC). (**a**) CD lesion involving entire digestive tract and (**b**) UC lesion located in large intestine and rectum.

**Figure 2:**
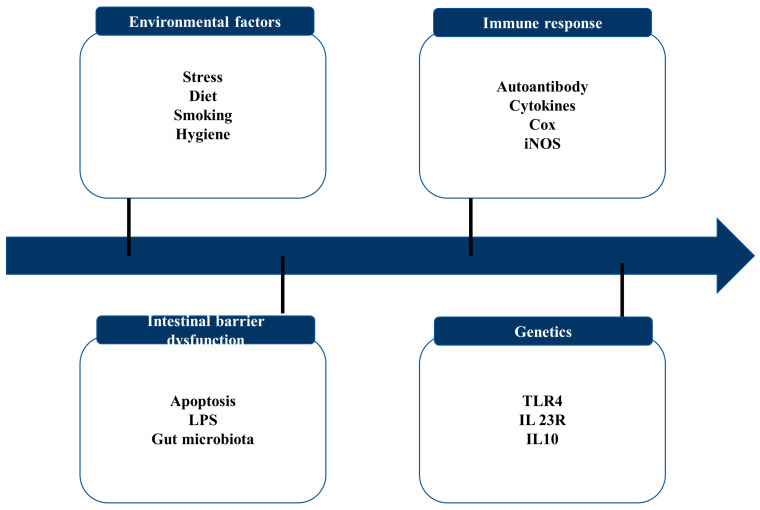
Key contributing factors to IBD.

IBD includes various types of idiopathic chronic inflammatory diseases. Due to their severity, frequency of complications, and mortality, Crohn’s disease (CD) and ulcerative colitis (UC) are considered the main disease categories in IBD (Figure 1) [4]. While studies have shown that the etiology of IBD is complicated, the key contributors include genetic predisposition, epithelial barrier abnormalities, dysregulated immunological responses, and environmental variables (Figure 2) [5]. The immunological dysregulation in IBD occurs due to the effector and regulatory cell dysfunction in innate immunity, leading to the uncontrolled release of soluble inflammatory mediators [6]. CD and UC result in the activation of T cells, but differ in terms of the differentiation and activation of T cell immunity. CD is described as a Th1 and Th17 condition with an increased production of IL-12, IL-23, IFN-ɤ, and IL-17, whereas UC is characterized as a Th2 condition with increased IL-12, IL-5, and IL-9 production [6]. Recently, much information has been gathered on malfunctioning innate immunity as the primary mechanism involved in IBD development. Innate immunity recognizes pathogen-associated molecular structures (PAMPs). These include NOD-like receptors (NLRs), mannose receptors, and Toll-like receptors (TLRs). The ongoing inflammatory process occurs from the inappropriate control of these signaling pathways during both the acute and chronic phases of intestinal inflammation, resulting in the pathogenesis of IBD [7]. Modern medical therapy, including anti-inflammatory drugs (e.g., corticosteroids), biological drugs (antibiotics, adhesion molecule antagonists), and immunosuppressants such as thiopurine and calcineurin inhibitors, are used to treat IBD [8]. However, severe adverse effects are prominent when using synthetic drugs. Several studies have reported that aminosalicylate causes severe headaches and nausea, while corticosteroids lead to obesity, hypertension, and diabetes [9]. Moreover, glucocorticoids have been proven to work best for short-term treatments, and their long-term application has been reported to cause moon face and weakened immunity [10]. Therefore, finding novel sources of medications to lessen the severe side effects of traditional therapy is crucial for treating the clinical symptoms of IBD.

Recent developments in understanding the pathogenesis of IBD have created opportunities for developing novel therapeutic approaches focused on therapeutic targets and involving safe and efficient natural compounds that can normalize the intestinal microflora, maintain clinical remission, and speed up the healing of intestinal mucus layers [11,12,13]. Marine seaweed serves as a significant bioresource of natural compounds with beneficial activities. Seaweed consists of high amounts of polysaccharides, proteins, and polyphenols. Polyphenols from marine seaweed have been studied for their therapeutic activities, such as anti-viral, antioxidant, and photoprotective activities [14,15,16]. Research on the potential for employing complex marine polysaccharides as medicinal agents has been explored extensively. In addition to serving as supporting elements for tissues and stores for nutrients in both plants and animals, polysaccharides have a wide range of physiological functions, such as immune regulation as well as anti-inflammatory, antioxidant, and anti-viral effects [17,18]. According to a significant amount of experimental evidence, algal polysaccharides and algae extracts appear to have anti-inflammatory and gastroprotective actions, making them extremely promising for treating and preventing gastrointestinal illnesses [19,20,21]. Algal polysaccharides are excellent therapeutic agents for treating intestinal inflammatory diseases such as IBD due to their resistance to the action of the gastric juice and enzymes of the host and their ability to serve as a fermentation substrate to beneficial intestinal microbes [22,23]. Therefore, the present study mainly focused on polysaccharides isolated from marine seaweeds and their usage for treating IBD.

## 2. Methodology

The following methodology section explains the methods used to conduct the review. For the identification of relevant studies, a comprehensive search of electronic databases, including PubMed, Scopus, and Web of Science, was performed. Keywords such as “seaweed”, “polysaccharides”, “inflammatory bowel disease”, “ulcerative colitis”, and “Crohn’s disease” were used. In addition, a manual search of the reference list of relevant studies was performed to identify additional studies. Studies that examined the effect of polysaccharides on IBD in human and animals were included in the review, while studies that focused on other seaweed components were excluded. The data were extracted from eligible studies, including study design, sample, intervention type, outcome, and statistical significance. The data were synthesized using a narrative approach, which involved summarizing the finding of each study and pattern recognition.

## 3. IBD Mechanism

IBD is marked by recurring chronic intestinal inflammation and a poorly understood pathophysiology. Current research has demonstrated the four pathogeneses of IBD, including intestinal flora disorders, immunological responses, environmental factors, and genetic vulnerability [24].

### 3.1. Intestinal Epithelium

The first line of defense of the body against invading pathogens is the intestinal barrier. The mucus secreted by epithelial cells protects the cells from invading pathogens by creating a physical barrier. The epithelial cells in the intestine renew rapidly, with the cells shedding onto the intestinal lumen. Stem cells proliferate in the intestinal crypt to compensate for the ongoing cell loss. A failure in this process leads to severe damage to the barrier, resulting in extreme intra-luminal antigen invasion and inflammation [25]. An epithelial cell abscission due to tight junction protein (TJ proteins) rearrangement is another negative effect leading to colitis. As reported by Martini et al., 2017, an increased expression of Myosin light chain kinase resulted in tight junction dysregulation and the loss of epithelial barrier function in mice [26]. The main factor causing reduced barrier function and the increased permeability of colonic mucosa is the abnormal TJ protein expression. This allows the entry of toxic substances and pathogen microbes into the gut, leading to IBD. The attachment of pathogenic bacteria to the intestinal epithelium reduces the intestine’s integrity by withdrawing occluding ZO-1 and claudin from tight junctions [27].

The beneficial intestinal microflora in the gut produces a series of secondary metabolites containing short-chain fatty acids (SCFA). These encourage mucus secretion by goblet cells and promote antimicrobial peptide production, increasing intestinal integrity. These processes eliminate the harmful bacteria in the gut, which leads to IBD [28]. The intestinal epithelium cells regulate the intestinal mucosal innate immune defense and microbiome balance. Moreover, they work as a center for internal and external signaling pathways. Individuals with IBD drop their tolerance to symbiotic bacteria and produce increased inflammatory responses [29].

### 3.2. Intestinal Microflora

The incidence and progression of IBD are closely correlated with the gut flora’s homeostasis [30]. Several reports have been published regarding intestinal microbiotas’ contribution to IBD [31]. The intestinal microenvironment is composed of bacteria infiltrating intestinal lymphatic tissues. The decrease in anti-inflammatory microbes, such as *Clostridium* and Bacteroides, and the increase in colitis stains, such as *Escherichia coli*, occur due to the imbalance and irregular changes in the abundance and diversity of intestinal bacteria [32]. Studies reported that in CD patients, *Veillonellaceae* and *Enterobacteriaceae* are abundant in their mucus [33]. The primary initiator of intestinal damage in the pathogenesis of IBD is the fast rise in the generation of free radicals induced by phagocyte infiltration and the imbalance of intestinal homeostasis caused by the damage to the antioxidant defense system [34]. Increasing reactive oxygen species (ROS) production is one of the critical factors in intestinal tissue injury and inflammation. The SCFA produced by intestinal microflora lowers the intestine’s pH, inhibiting pathogenic bacterial growth and allowing the proliferation of beneficial bacteria. This results in the improvement of the homeostasis of intestinal flora [35].

### 3.3. Immune System

The intestine’s immune system tolerates dietary antigens while protecting the mucosal surface from infections and injuries. This necessitates a careful balance between regulatory T cells and effector T cells, and it is believed that the disruption of this equilibrium leads to CD and UC development [36]. The immune system depends heavily on helper T cells (Th), which include Th1, Th2, Th17, and regulatory T cells (Treg). In mice with UC, it has been discovered that Treg cell deficiency worsens IBD symptoms. It was proposed that the Th1 response regulates CD, while UC is tightly linked to an atypical Th2 response accompanied by the IL-5 and IL-3 cytokines [37]. Apart from this, the stimulated Th17 cells stimulate the production of IL-17, GM-CFS, IFN-ɤ, TNF-ɑ, and IL-22 from the inflamed intestinal tract. Although Treg cells serve a crucial anti-inflammatory and immune regulatory role, Th17 cells exert a pathogenic effect [38]. Studies have shown that transferring the two Treg cells CD4+ and CD25+ into colitis-affected mice can lessen the infiltration of inflammatory cells in the lamina propria and return the intestinal wall to its normal state, reversing the inflammatory response and healing the colitis [39].

The macrophages gather around the IBD lesions in the intestine and secrete anti-inflammatory cytokines following biological effects. The pro-inflammatory cytokines IL-1, IL-12, IL-23, TNF-ɑ, and many others are secreted by M1-type macrophages, and they also break down the NO and ROS that L-arginine produces. The Th1 and Th17 cell-mediated immune responses are overexpressed by inducible nitric oxide synthase (iNOS), which results in inflammatory injuries in the intestinal tissues [40]. Gut microbes digest lipopolysaccharide (LPS) and release it into the blood through the intestinal barrier. LPS activated the Toll-like receptor 4 (TLR-4) pathway, promoting pro-inflammatory cytokine production. The functions of TLR4 include initiating an innate immune response in the early stages of pathogen infection, promoting the expression of pertinent immune molecules, and stimulating the maturation and activation of immune cells, which bridges the gap between innate immunity and adaptive immunity [41]. Since numerous signaling pathways and cytokines are simultaneously stimulated in IBD, a multi-target treatment is more likely to reverse the illness successfully.

## 4. Marine Polysaccharides

Seaweeds are mainly categorized as brown, green, and red. Polysaccharides obtained from seaweeds vary greatly depending on their taxonomic classification [42,43]. Seaweed’s structural polysaccharides are the most abundant, and their composition changes with species and environmental factors [44].

### 4.1. Brown Seaweeds

Brown seaweeds contain alginates and fucoidan, proven clinically and experimentally to possess pharmacological effects. Fucoidan is a sulfated polysaccharide consisting mainly of ʟ-fucose and sulfate ester groups [45]. Apart from fucose, it contains glucose, galactose, xylose, and uronic acids [46]. Fucoidans are mainly derived from the cell wall matrix of many kinds of brown algae, serving important defensive and structural purposes [47]. The ionic nature of fucoidans (negative charge) resulting from the presence of sulfate in both the second and fourth carbon positions makes them more suitable for applications in pharmaceutical technology. It allows them to form complexes with other charged molecules [48]. The low toxicity, biodegradability, and biocompatibility of fucoidans make them safe as food ingredients [48]. Alginic acids are found in brown seaweeds, such as kelp, Gulfweed, Ascophyllum, and macroalgae. The alginic acid in the cytoplasm is vital in strengthening the cell wall [49]. It is found in 18–40% of the plant’s total mass [50]. Similarly to fucoidan, alginic acids are non-toxic, biocompatible, and non-immunogenic. Apart from alginate’s ability to be a stabilizing agent, alginates are used in the biomedicine and food industries [51].

Laminarin is another underexploited polysaccharide found in brown seaweeds. Laminarins typically consist of low-molecular-weight (5–10 kDa) β-glucans with β-(1-3)-linked d-glucose residues [52,53]. These polysaccharides have been reported to possess distinct therapeutic roles, such as anti-inflammatory, antioxidant, anti-apoptotic, and anti-coagulant effects [54,55,56,57].

### 4.2. Green Algal Polysaccharides

Ulvan is a water-soluble sulfated polysaccharide found in green algae, which acts as a structural component of an algal cell wall. The structure represents a sequence of disaccharide units comprising two different types of aldobiuronic acid, ulvanobiuronic acid 3- sulfate type A and type B [58]. The structure of ulvan disaccharide moieties is similar to that of animal connective tissue glycosaminoglycans found in the extracellular matrix [59]. Ulvan has shown considerable biological activity in both animal and plant systems. Ulvan has been reported and proven to contain anti-coagulant, immunomodulating, anti-cancer, antioxidant, anti-viral, anti-hyperlipidemic, and anti-inflammatory activities [60,61,62,63].

### 4.3. Red Algal Polysaccharides

In contrast to the other two kinds of green and brown seaweed, which include polysaccharides, red seaweed is an essential source of many beneficial bioactive compounds. The main structural component of red algae is sulfated polysaccharides. Carrageenan, agar, and xylan are common polysaccharides found in red algae. The protein content of red algae is around 10–50% of its dry weight [64]. Carrageenan is an anionic sulfated polysaccharide commonly found in red algae, such as *Chondrus*, *Gigartina*, *Hypnea*, and *Eucheuma* [65,66]. It is a component of the outer cell wall and intracellular matrix and is composed of linear polysaccharide chains with sulfate half-esters attached to the sugar units. The three general forms of carrageenan are kappa, lambda, and iota. This classification is based on the availability of 3,6- anhydrogalactopyranose and the allocation of sulfate groups on the main structure [67]. Carrageenans are the algal polysaccharides most thoroughly researched regarding their toxicity, pyrogenicity, allergenicity, food safety, and medicinal usage [68]. Carrageenans are potential immunomodulating, antioxidant, anti-inflammatory, and anti-viral agents [69,70].

## 5. Therapeutic Targets of Algal Polysaccharides against IBD

### 5.1. Intracellular Adhesion Molecules

Selectins, integrins, and cadherin-like adhesion molecules are vital in migrating leukocytes in inflammatory areas, and these are potential therapeutic targets for chronic intestinal inflammation [71]. Selectin is bound to mannose, and fucose slows the movement of leukocytes and platelets to the endothelium surface, improving transendothelial transmission. The physiological effects of the interaction between fucoidan and selectins have potential therapeutic benefits. In 1999, Semenov et al. reported that rats with experimental peritonitis received fucoidan intravenously after receiving peptone intraperitoneally, significantly suppressing the neutrophils discharge into the abdominal cavity [72]. According to the authors, the interaction between fucoidan and P-selectin inhibits inflammation at an early stage of its development. Another study reported that the intravenous administration of fucoidan led to a reduction in colonic mucosal injury and crypt damage in dextran sodium sulfate (DSS)-induced mice. They further stated that this effect was due to the reduction in abolishing venular leukocyte rolling [73]. These findings demonstrate that sulfated polysaccharides, which exhibit the characteristics of glycosaminoglycan mimics, block adhesion molecules and restrict leukocyte migration, providing beneficial relief for inflammatory bowel illnesses.

### 5.2. Intestinal Epithelial Cells

Reducing the efficacy of the epithelial barrier is believed to have a significant role in the etiology of IBD. The epithelial barrier comprises an intact epithelial monolayer and tight junctions by which the membranes of the neighboring cells are brought together. They are cross-linked with two proteins, namely occluding and claudin [74]. Previously reported studies have proven that tight junction abnormalities and excess paracellular permeability result in the increased stimulation of antigens, leading to the development of IBD. Therefore, the maintenance of remission in people with IBD or the prevention of illness in at-risk persons may benefit from restoring intestinal barrier function [75]. A study on the potential of fucoidan against H_2_O_2_-induced epithelial barrier function damage reported that the treatment of fucoidan dose-dependently increased the transepithelial resistance and protected the intestinal epithelium from paracellular permeability. Fucoidan treatment was also reported to increase claudin-1 expression. According to the authors, fucoidan, which boosts epithelium protective function and encourages epithelial regeneration, may be a suitable therapy for managing IBD [76]. A group of researchers who investigated the anti-inflammatory activity of *Laminaria japonica* against intestinal inflammation reported that the seaweed improved the intestinal barrier function and prevented tight-junction-related protein inhibition. It also downregulated the cells’ IL-6 and nitric oxide levels [77].

A study in 2014 conducted by Wu et al. reported that bacterial-derived lipopolysaccharides cause intestinal barrier function defectives [78]. They reported that sulfonate fucoidan nanoparticles coated with berberine inhibit the redistribution of tight junction proteins ZO-1 and ameliorate LPS-induced intestinal epithelial tight junction disruption. In dietary-fiber-deficient mice, feeding them *Scytosiphon lomentaria* was proven to induce epithelial cell layer integrity and the loss of goblet cells as well as inhibit inflammatory cell infiltration, leading to the inhibition of colon damage [79]. The illustrations of their results demonstrate how algal polysaccharides might influence intestinal health by reducing intestinal inflammation and improving barrier integrity by partially controlling dense substances and related proteins (claudine, occludine).

### 5.3. Pro-Inflammatory Cytokines

It has been undeniably proven that cytokine responses are essential components for managing the inflammatory processes underlying IBD [11]. In addition to causing intestinal inflammation and diarrhea in IBD, cytokines control systemic effects and extra-intestinal disease symptoms (such as arthralgia or arthritis). Moreover, cytokines appear to be a significant factor in developing IBD complications, such as intestinal stenosis, fistula formation, and colitis-associated neoplasias [80]. The cytokine spectrum, including pro-inflammatory cytokines IL-6, IL-12, IL-23, and IL-21 as well as anti-inflammatory cytokines such as IL-10 and TGF-β, has been identified as possible novel targets for treating intestinal inflammation in studies using tissues from individuals with IBD and animal models of IBD [81,82]. Among the targets, IL-17 has been recognized as the main pathogenic factor stimulating IBD [83]. The fact that TNF blocking is now a popular treatment option for IBD in clinics emphasizes cytokines’ crucial function in IBD [84].

Due to the modulation of several pro-inflammatory processes and mediators, including the control of the gene expression of pro- and anti-inflammatory cytokines associated with UC, brown algae polysaccharides are a potential therapeutic option for patients with IBD [18,85]. A study conducted on laminarin and fucoidan on the pathology and inflammation in pigs resulting due to DSS showed that a combination treatment prevents weight loss and diarrhea. Moreover, the expression of IL-6 was also downregulated with the co-treatment of laminarin and fucoidan [86]. In 2018, Sudirman et al. evaluated the potential of *Eucheuma cottonii* polysaccharides against the inflammatory response in mice induced with DSS [87]. The polysaccharides were orally treated, ameliorating the weight loss in DSS-induced mice while decreasing their colon weight. The administration of polysaccharides was reported to down-regulate the expression of pro-inflammatory cytokines TNF-ɑ, IL-1β, IL-6, and IL-10.

In vitro studies using the colon epithelial cell line CMT-93 stimulated by lipopolysaccharides and in vivo studies using mice with chronic colitis brought on by sodium dextran were conducted by Matsumoto et al. in 2004 to examine the effects of different types of fucoidans as inhibitors of IL-6 production. It was demonstrated that the fucoidans from *Kjellmaniella crassifolia* and *Cladosiphon okamuranus* Tokida reduced IL-6 production in CMT-93 cells and decreased NF-kB nuclear translocation. Fucoidan from *Cladosiphon okamuranus* also decreased the expression level of IL-6 mRNA in mouse epithelial cells compared to animals fed a regular diet, while boosting the synthesis of IL-10 and TGF-β and inhibiting the synthesis of IFNɤ and IL-6. A study on β-glucan obtained from *Laminaria hyperborean* and *L. digitata* proved that the expression of Th-17-associated cytokines, such as IL-17a, IL-17F, and IL-22, decreased. Moreover, the expression of IL-23R and IL-6 receptors also decreased. No alterations of T regulatory cell (Treg)-related targets were observed in the study [88]. In 2015, Lean et al. reported the potential activity of a fucoidan–polyphenol complex obtained from *Fucus vesiculosus* on a DSS-induced mouse model of acute colitis [89]. According to their results, the oral administration of polysaccharides considerably reduced diarrhea, fecal blood loss, weight retention, and other colitis symptoms compared to the colitis group that did not receive treatment.

Furthermore, in mice given oral fucoidan, the weights of their colon and spleen were likewise much lower, indicating lessened inflammation and edema. The reduced production of inflammatory cytokines in the colin tissues resulted in reduced macroscopic alterations. Notably, the intraperitoneal administration of deproteinized fucoidan increased the animals’ condition and the expression of pro-inflammatory cytokines in their colon tissues. The study proposed the usage of fucoidan as an oral drug to decrease inflammation and preserve the integrity of the intestinal epithelium.

### 5.4. Intestinal Microbiome

The most important development in IBD research over the past ten years has been the idea of dysbiosis or changed gut flora. Modern molecular and genetic diagnostic methods have revealed the deficits of innate immunity and unique alterations in the gut microbiota of IBD patients [90]. IBD has started to be understood as an immunodeficiency disease, with compromised microbiota significantly contributing to the long duration of inflammation. In light of this, altering the intestinal microbiome through antibiotics, prebiotics, and probiotics is a promising approach for preventative and therapeutic IBD intervention [91,92,93,94].

The most recent definition of prebiotics includes “substrates that host bacteria employ specifically to produce health benefits”. Three requirements must be met for substances to be considered prebiotics: they must be resistant to digestion and the action of upper gastrointestinal tract enzymes; they must be fermented by colon microflora; and they must also be a selective substrate for the growth of beneficial bacteria and have local or systemic effects that are advantageous to the host [93]. According to experimental and clinical studies, prebiotics inhibit potentially pathogenic bacteria, lessen mucous membrane inflammation, and lower the risk of subsequent clinical relapses of IBD. They also promote the growth of probiotic strains in the intestine and inhibit potentially pathogenic bacteria [95,96,97]. As they may be added to food, feed, or taken as tablets, algal polysaccharides and oligosaccharides have an advantage over other sources for the manufacture of prebiotics. These dietary fibers are different from fibers of terrestrial origin in terms of their chemical and physicochemical characteristics. Their quantity is more significant than that in most fruits and vegetables and varies from 33 to 50 g per 100 g of algae [98]. Several polysaccharides are currently used as prebiotics and in IBD treatment [99,100]. Data on the potential for fucoidan to be fermented by gut bacteria are conflicting. Sulfated polysaccharides have been shown to have prebiotic action in vitro, although there is an apparent delay in using them as prebiotics. This situation can be due to the need for clinical trials and insufficient in vivo research on the prebiotic potential of algal polysaccharides [101,102].

The beneficial bacterial species in the intestine are known as “probiotics”, which include *Bifidobacteria*, *Lactobacillus*, and *Faecalibacterium* spp. These bacteria possess immune regulatory potentials, such as *Lactobacillus* up-regulating the dendritic cell maturation, resulting in the production of IL-12, IL-18, and IL-23 and contributing to the Th1 responses. They also produce IL-4 and IL-10, contributing to the Th2 responses [103,104]. *Bifidobacteria* can boost IL-10 release in DC and decrease IFN production via activated CD4+ T cells, while *Faecalibacteria* increases the IL-10 production in DC and reduces IFN-ɤ production via activated CD4+ T cells [105]. In 2012, Kuznetsova et al. showed that cultivating bifidobacteria on a nutrient medium enriched with *Fucus evanescens* fucoidan increased their growth and the accumulation of biomass [106]. Likewise, polysaccharides from *F. evanescens* were also proven to have prebiotic activity on B. bifidum by testing in vivo a drug dysbacteriosis mice model after one month of treatment [107]. In 2016, Shang et al. reported that the oral treatment of fucoidan isolated from *Ascophyllum nodosum* and *L. japonica* increased the abundance of *Lactobacillus* and *Ruminococcacea*-like beneficial bacteria.

Moreover, dietary fucoidan also dramatically lowered the antigen load and the inflammatory response in the host, as seen by the decreased blood lipopolysaccharide-binding protein levels, by maintaining a more balanced composition of gut microbiota [108]. The in vitro fermentation of sulfated polysaccharides from *Enteromorpha prolifera* and *L. japonica* was reported to be effective as prebiotics in a study conducted by Kong et al. in 2016. The fermentation increased SCFA, such as acetic, butyric, and lactic acids, while modulating the microflora balance in the gut [109].

The information mentioned above demonstrates that algae polysaccharides have tremendous potential for use as prebiotics in IBD and can achieve health effects, such as regulating the composition and functions of microbiota, lowering the pH in the colon lumen, preventing intestinal colonization by pathogens, and reducing the production of reactive oxygen species, which are a source of energy for colonocytes and activate free fatty acid receptors. More health benefits of algal polysaccharides against IBD are given in Table 1.

## 6. Algal Polysaccharides in Drug Delivery Systems

To treat IBD, medications are desired to be taken orally to reach the colon [122]. It is challenging to provide oral medications to the colon at the distal end of the gastrointestinal system due to physiological issues, biochemical obstacles, and environmental barriers, such as those brought on by mucus and the epithelium [123]. Several polymer designs (including linear and branched leading chains) and polymer combinations are currently suggested as carrier systems [124]. Certain polysulfated fucoidan chains can bind to TLR4, CD14, scavenger receptors, the mitogen-activated protein kinase receptor, and receptors specialized for mannose, fucose, galactose, and N-acetylglucosamine residues. They can also influence the effects of signaling molecules on cells [125]. Sulfated polysaccharides can therefore be utilized as precise recognition signals to target immune system cells and encourage drug accumulation in the inflamed gut.

Polysaccharides have the potential to not only alleviate inflammatory bowel disease on their own but also to act as a drug carrier for additional potent medications that can be employed to treat IBD specifically. Other beneficial factors include the capacity to chemically modify polysaccharides to have the following properties: high stability, biodegradability, safety, non-toxicity, hydrophilicity, and gel-forming. Recent preclinical investigations have demonstrated the promise of algal polysaccharide-based nanoparticle drug delivery systems as future therapeutics for IBD [126]. Most frequently, chitosan, carrageenan, and fucoidan have been indicated as matrix materials in the creation of nanoparticles. The sulfate group of polysaccharides interacts with the amino group of chitosan to create nanoparticles and regulate medication release [115]. Moreover, the fucoidan–chitosan nanoparticles’ pH-responsive profile prevents the gastrointestinal tract’s acidic conditions from degrading them and permits medicine to be absorbed in the gut. For this reason, the oral administration of active medicinal substances using fucoidan and chitosan nanoparticles has been thoroughly explored [126,127].

In order to create a compound with intense anti-inflammatory action and minimal toxicity, in 2017, Zhu et al. coated selenium nanoparticles with a polysaccharide from the green alga *Ulva lactuca* [128]. The complex was proven to have high anti-inflammatory action and minimal toxicity. In mice with DSS-induced acute colitis, supplementation with ULP-SeNPs significantly reduced body weight loss and colonic inflammatory damage, among other adverse effects. The reduced CD68 values in the colon tissue sections proved that the ULP-SeNPs reduced macrophage infiltration. The plasma levels of TNF-ɑ and IL-6, COX-2, and iNOS were reduced in complex-treated animals compared to control animals. The ULP-SeNPs acted by blocking the nuclear translocation of NF-κB, which activates these pro-inflammatory cytokines. These studies demonstrate the efficacy of drug delivery methods based on polysaccharides from seaweed for IBD therapy. However, more thorough research on the patterns of material accumulation in the intestinal tissues is required to implement this approach in the treatment of IBD successfully.

## 7. Conclusions

Due to its serious outcomes, IBD has become a popular research topic in recent years. Algal polysaccharides contain valuable anti-inflammatory, antioxidant, anti-tumor, and other physiological activities, which are especially useful in preventing IBD. Polysaccharides isolated from seaweed can inhibit IBD directly as well as indirectly. Due to their structural characteristics, seaweed polysaccharides have a pleiotropic effect that allows them to influence therapeutic targets for IBD, such as inflammatory cytokines, adhesion molecules, intestinal microbiota, and intestinal epithelial cells. Algal polysaccharides also have a lot of promise for creating drug delivery systems because of their physicochemical characteristics, which allow for interactions with many substances, including medicines, proteins, and other polymers. However, the bulk of the investigations into using algal polysaccharides in IBD have been conducted ex vivo or in trials on animals. While preclinical studies have shown promising results, there is a lack of clinical trials investigating their efficacy in humans with IBD. Clinical trials are necessary to establish the safety and effectiveness of algal polysaccharides in treating IBD. Moreover, most studies investigating algal polysaccharides have compared them to a placebo or control group. More research is needed to compare their effectiveness to standard treatments in IBD, such as anti-inflammatory drugs or immunosuppressive agents. Unquestionably, the use of algal polysaccharides in medicine will increase annually as science advances and the possibilities for generating standardized medicines based on these chemicals increase. The shortcomings of the present review include limited data availability and the exclusion of certain studies due to language and publication biases. However, to improve the review, the search criteria were expanded and multiple databases were used.

## Figures and Tables

**Table 1:** Algal derived-polysaccharides and their protective effect and mechanism on intestinal health.

Source	Polysaccharide Type	Inducer	Model	Function	Reference
*Laminaria japonica*	Fucoidan	Cefoperazone	Mice	Reduces H_2_O_2_-induced paracellular permeability. Up-regulates the expression of claudin-1, claudin-2, and occludin.	[76]
*Sargassum fusiforme*	Crude sulfated polysaccharide	-	Bacteria	Increases the availability of *Faecalibacterium*, *Phascolarctobacterium*, *Ruminococcaeceae*, and *Lactobacillus*.	[110]
*Laminaria japonica*	fucoidan	-	Mice	Increases *Ruminococcaceae* and *Lactobacillus*.	[111]
*Undaria pinnatifida*	Fucoidan	Hight-fat diet	Mice	Reduces weight gain and fat accumulation, as wel as restores the normal level of *Firmicutes* and *Bacteriodetes* in mice fed high-fat diets.	[112]
*Acaudina molpadioides*	Fucoidan	Cyclophosphamide	Mice	Alleviates inflammation, increases protein expression in tight junctions and the abundance of *Coprococcus*, *Rikenella*, and *Butyricicoccus* bacteria.	[111]
*Sargassum thungbergii*	Crude polysaccharide	-	-	Increases intestinal beneficial bacteria.	[113]
*Cladosiphon okamuranusv*	Fucoidan	LPS	Zebrafish	Down-regulates IL-1β pro-inflammatory gene expression.	[114]
*Gracilaria biridae*	Sulfated polysaccharide	Trinitrobenzenesulfonic acid (TNBS)	Rat	Reduces TNBS-induced intestinal damage.	[115]
*c*	Sulfated polysaccharide	Acetic acid	Mice	Inhibits colonic inflammation by reducing the wet weight of colon, reduces MPO enzyme activity, GSH consumption, and pro-inflammatory cytokine production.	[116]
*Ecklonia cava*	Fucoidan	LPS	zebrafish	Inhibits ROS and NO production.	[117]
*Hypnea musciformis*	Sulfated polysaccharides	TNBS	Rats	Reduces inflammatory responses by reducing neutrophil migration, decreases cytokine levels, and downregulates oxidative stress and intestinal damage	[118]
*Gracilaria lemaneiformis*	polysaccharides	Dextran sulfate sodium (DSS)	Mice	Improves DSS-induced colitis symptoms, alters intestinal microbes, increases SCFA production, and downregulates CCL25/CCR9,TGF-β1 levels.	[119]
*Blidingia minima*	Polysaccharides	DSS	Mice	Repairs colon dysfunction and colonic morphology, improves the expression of tight junction proteins, and decreases pro-inflammatory cytokine levels.	[120]
*Eucheuma cottonii*	Polysaccharide	DSS	Mice	Protects against weight loss, decreases the colon weight per length ratio, decreases pro-inflammatory cytokine levels, increass IL-10 production, and reduces colonic damage.	[87]
*Ulva pertusa*	Sulfate ulva polysaccharide	DSS	Mice	Decreases colon shortening and colonic tissue damage.	[121]

## Data Availability

Not applicable.
